# Comparison of the Fatality of Some of the Diseases of London, Now and in the Seventeenth Century

**Published:** 1860-05

**Authors:** 


### COMPARISON

### OF THE FATALITY OF SOME OF THE DISEASES OF LONDON NOW AND IN THE SEVENTEENTH CENTURY.

The Medical *Times and Gazette* copies the following from the report of the Registrar General: The diseases were not always distinguished accurately; but by putting them in groups any fallacy from this source will be obviated, and the decrease of some of the worst forms of mortal disease will be placed beyond doubt. To render the comparison easy, the number living is taken to be the same in two periods, 100,000 in 1660 and 1679 and in 1859. The annual deaths by small-pox were 357 in the first period, 42 in the second period; by measles 40 and 47 in the two periods. Medical science was imperfect, and the science existed in that century was very imperfectly applied. Croup and scarlatina were not generally recognized, but were confounded with measles and fever. The mortality by fever, continued or remittant, and ague was at the rate of 749 and 59 in the two periods; or including scarlatina, croup and quinsy, the mortality was 759 and 227. Thus a person was in four times as much danger of dying of these diseases at the Restoration as a person living in London now. Women are not yet entirely exempt from peril in child-bearing; the mortality of this disease is now 17, it was then 86. Again a few (8) in 100,000 die now of dysentety; then out of the same number, 753 died annually of that disease. By diarrhœa, a milder form of disease, 11 died then, 120 die now; cholera was fatal in 1859 to 7, and in the 20 years (1660–79) to 130 annually. Syphilis was twice as fatal as it is, the numbers being 21 and 12. Scurvy and purpura bear testimony to the imperfect nutrition of the population; the annual deaths were then 142, and are now 2. Vegetables, fruit, and fresh meat, could with difficulty be procured in Winter. Worms, and all parasitic creatures that crawl over, bite, and prey on the body of man, were prevalent; 10 deaths were ascribed to worms. Dropsy, a result and sign of scurvy and fever, was exceedingly fatal; 298 died of that disease then, and 26 now. Apoplexy, paralysis, epilepsy, affections of the brain, and suicide, are more fatal now, according to the returns, than they were in the proportion of 57 then to 151 now. Consumption and diseases ot the breathing organs were uncommonly fatal; 1079 then and 611 now are the figures of the mortality. Diseases ot the digestive organs were fatal then and now in the proportion of 146 and 95. Stone and diseases of the urinary organs are not now as fatal as they were then; the deaths being 21 and 30. Children were rapidly cut down; of convulsions and teething, 1175 died then, 136—too many—now. —*Med. and Surg. Reporter.*

